# Forebrain networks driving brainstem pain modulatory circuits during nocebo hyperalgesia in healthy humans

**DOI:** 10.1097/j.pain.0000000000003604

**Published:** 2025-05-29

**Authors:** Lewis S. Crawford, Sora Yang, Noemi Meylakh, Leana Sattarov, Alister Ramachandran, Vaughan G. Macefield, Kevin A. Keay, Luke A. Henderson

**Affiliations:** a School of Medical Sciences (Neuroscience), Brain and Mind Centre, University of Sydney, Sydney, Australia; b Westmead Hospital Pain Management Centre, New South Wales, Australia; c Department of Neuroscience, Monash University, Victoria, Australia

**Keywords:** Midbrain periaqueductal gray matter, Anterior cingulate cortex, Dorsolateral prefrontal cortex, Connectivity, Dynamic causal modelling

## Abstract

Prior experiences, conditioning cues, and expectations of improvement are essential for nocebo hyperalgesia expression. The neural circuits that communicate with brainstem pain modulatory nuclei during nocebo hyperalgesia responsivity are underexplored. In this study, we employed a classical conditioning and expectation model in 25 healthy human participants and measured brain activity using ultra-high field functional magnetic resonance imaging. We assessed forebrain activity changes as well as noxious stimulus-independent and -dependent connectivity changes with the lateral midbrain periaqueductal gray matter (lPAG). We found hyperalgesia-related signal increases in the orbitofrontal cortex, insula, and amygdala. In addition, we found stimulus-dependent lPAG connectivity changes with the orbitofrontal, anterior cingulate, and dorsolateral prefrontal cortices and stimulus-independent lPAG connectivity with the anterior cingulate cortex, dorsolateral prefrontal cortex, and nucleus accumbens during hyperalgesia. Whilst these connectivity changes are all associated with hyperalgesia, dynamic causal modelling analysis revealed that the dorsolateral prefrontal cortex was principally responsible for driving the lPAG. Overall, our results show that there is a complex relationship between forebrain activation and connectivity with brainstem pain modulation circuitry that results in the behavioural expression of nocebo hyperalgesia.

## Abstract 2

The forebrain circuits that regulate pain modulatory circuits during hyperalgesia are unknown. We identify forebrain activity and forebrain-brainstem connectivity that underpins nocebo hyperalgesia.

## 1. Introduction

Nocebo hyperalgesia is a powerful phenomenon in which an inert substance or visual cue provokes expectations,^24^ environmental associations,^29,32,44^ or provides a conditioning signal,^27^ to evoke a significant increase in the perceived intensity of pain. It is well known that in a clinical setting, simply disclosing to an individual the potential to experience higher pain can itself produce negative expectations and increased pain intensity.^4,16,42^ Indeed, by recognising the impact of interpersonal communication, healthcare providers can mitigate nocebo effects in pain medicine. However, the pervasive nature and coincidental elicitation of this phenomenon in clinical practice has led to considerable interest in better understanding nocebo's neural determinants.

Given the cognitive nature of nocebo effects, it is assumed that nocebo hyperalgesia involves the recruitment of descending projections from higher-order brain regions such as the prefrontal, insular, and cingulate cortices to midbrain pain-modulating centres such as the lateral periaqueductal gray matter (lPAG).^14,29,48^ Consistent with this hypothesis, functional magnetic resonance imaging (fMRI) studies have shown that nocebo hyperalgesia is associated with activity changes in the thalamus, amygdala, insula, and cingulate cortices.^20,25,35^ Furthermore, in a recent ultra-high field fMRI study, we demonstrated that nocebo hyperalgesia is associated with signal changes in the well-described brainstem pain modulatory circuit: the lPAG–rostral ventromedial medulla (RVM) axis.^11^ Critically, in the same study, we reported that placebo analgesia was also associated with signal changes in this brainstem circuit, albeit in an opposing direction.

Experimental animal studies have shown that lPAG sensitivity is regulated by descending subcortical inputs^13,15,23,49^ and recently showed that placebo analgesia responsivity is associated with altered descending connectivity onto the lPAG.^10^ More specifically, it was shown that functional connectivity between the lPAG and both the rostral anterior cingulate cortex (ACC) and hypothalamus were associated with placebo analgesia responsivity and that this was independent of noxious stimuli. Furthermore, noxious-stimulus-driven lPAG connectivity with the ACC and between the nucleus accumbens (NAc) and ACC were also associated with placebo responsivity. These data raise the possibility that similar modulation of the lPAG is also important in the expression of nocebo hyperalgesia, particularly descending inputs from areas such as the ACC, NAc, and hypothalamus, as is the case for placebo analgesia.

The overall aim of this investigation is to use ultra-high field fMRI to define forebrain activation and lPAG connectivity patterns during nocebo hyperalgesia. We hypothesise that consistent with previous studies, nocebo hyperalgesia will be associated with signal intensity changes in the thalamus, amygdala, insula, and ACC. In addition, we hypothesise that consistent with previous placebo analgesia results, stimulus-independent lPAG connectivity with the ACC and hypothalamus and stimulus-dependent lPAG connectivity with the ACC will be associated with nocebo hyperalgesia responsivity. Our data will provide a comprehensive view of nocebo hyperalgesia activation patterns as well as descending influences on the PAG-RVM pain modulatory circuit.

## 2. Methods

### 2.1. Ethics

All experimental procedures were approved by the University of Sydney Human Research Ethics Committee and satisfied the Declaration of Helsinki, with the exception of registration in a database. Written informed consent was obtained from participants at the commencement of this study. Participants were also provided with an emergency buzzer while inside the scanner so that they could stop the experiment at any time. At the conclusion of testing, participants were informed both verbally and through a written statement of the necessary deception and true methodology of the experiment and were invited to seek clarification of what they had just experienced.

### 2.2. Participants

Twenty-five healthy control participants were recruited for this study (12 men, 13 women; mean age, 22.7 ± 0.7 years [±SEM]; range 19–33 years). Exclusion criteria consisted of standard MRI contra-indications (ie, ferrous metallic implants, current or suspected pregnancy, pacemaker, etc.), any current chronic pain diagnoses or pain lasting longer than 3 months, and being below the age of 18. Power analysis conducted within G*Power 3.1^17^ using a previous brain imaging study identifying greater behavioural nocebo hyperalgesia relating to midbrain periaqueductal gray activity revealed a total sample size of 24 (12 per group) would be necessary to reveal similar effect sizes with 90% power (ԁ = 1.25; α = 0.9).^43^ Before beginning this study, participants completed a data sheet recording current medication(s), and any alcohol or caffeine ingested in the 24 hours before testing. Participants received reimbursement for travel costs and time lost due to travel to take part in this study in the form of $100 Australian dollar gift cards for each day of participation. Brainstem imaging results from this study have been published previously,^11^ with the current investigation focussing on forebrain changes.

### 2.3. Experimental design

**Figure 1.**
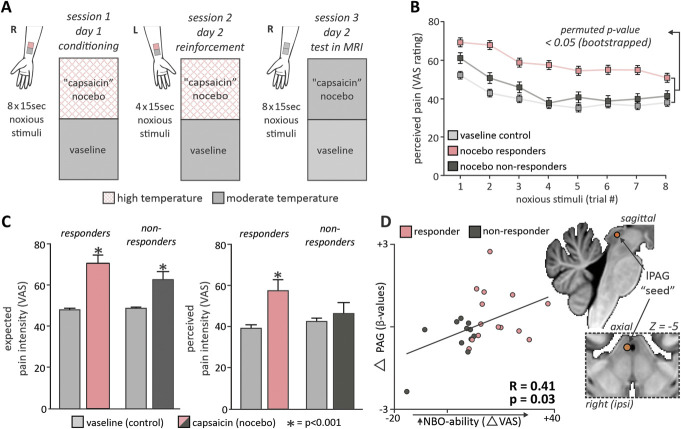
Experimental protocol, within-group behavioural findings, and midbrain periaqueductal gray involvement in nocebo hyperalgesia. (A) Conditioning and reinforcement phases involved a deceptively applied higher temperature to a nocebo “capsaicin” compared with a control vaseline cream site. During a test phase, whilst collecting fMRI series, both vaseline and “capsaicin” sites on the right forearm received identical stimulus temperatures, with differences in perceived pain encoding a participant’s nocebo hyperalgesia response. (B) Plots of mean pain intensities during each stimulus period in the vaseline and nocebo “capsaicin” series. Nocebo responders were classified as those with a significant increase in perceived pain during the stimulation of the capsaicin (red line) relative to vaseline (light gray line) cream. (C) Plots of mean ± SEM expected and perceived pain in nocebo responder (n = 14) and nonresponder (n = 11) groups. Note both groups expected a pain increase but only responders subsequently rated the pain intensity greater during the “capsaicin” compared with vaseline scans (*P < 0.001; paired *t* test). (D) The location of a 1-mm radius sphere “seed” in lateral midbrain periaqueductal gray matter (lPAG). Signal intensity changes were extracted from this seed to confirm the effect of greater nocebo hyperalgesia relating to greater PAG signal change (capsaicin mean β-value − control mean β-value).

This study included 3 sessions occurring on 2 successive days: a conditioning session on day 1, and a reinforcement and MRI scanning session on day 2 (Fig. 1A). Throughout this study, noxious stimuli were administered to the volar surfaces of participants' left and right forearms using a 3 × 3-cm MR-compatible Peltier element thermode, which delivered a heat stimulus at a preprogrammed temperature using a Thermal Sensory Analyzer (TSA-II) (Medoc, Ramat Yishai, Israel). Each stimulus lasted 15 seconds, including a ramp-up period (4°C/sec), a plateau period at a noxious temperature, and a ramp-down period (4°C/sec). Each stimulus was separated by a 15-second interstimulus interval at a nonpainful temperature of 32°C. Throughout conditioning, participants rated their pain online using a horizontal 10-cm visual analogue scale (VAS), ranging between 0 and 100, where 0 was described as “no pain” and 100 as “the worst pain imaginable.” During scanning, participants used an MR-compatible button box to continuously report their pain perception. The VAS scale was shown on a reflected digital screen at the end of the magnet bore, and participants controlled the position of a slider to report their pain continuously by holding the left (moved slider towards zero) or right (moved slider towards 100) buttons with their left middle and index finger.

Before commencing the experiment, all participants completed several questionnaires that showed relations with the pain percept or expectancy modulation. These included the behavioural activation and inhibition scales (BIS/BAS)^6^; the state and trait versions of the state-trait anxiety inventory^40^; the life orientation test revised form^33^; and the pain catastrophizing scale.^41^

### 2.4. Conditioning

Session 1 was conducted outside the MRI and consisted of 2 rounds of a response conditioning protocol. Participants were first informed both verbally and through a written statement that this study was designed to investigate the modulatory effects of a topical pain enhancer containing “capsaicin,” which had been shown to increase pain in some individuals. A second control cream was stated to be purely vaseline and described as being necessary to evaluate typical pain responses. In reality, both creams contained solely vaseline and only differed in colour and their described properties. We then conducted a “determination of moderate pain” test, where 10 randomised stimuli ranging from 44 to 48.5°C in 0.5°C intervals were delivered to the volar aspect of the left forearm. Participants were informed that we were interested in recording a temperature which elicited a moderate subjective pain response (40-50 VAS rating) and that this temperature would be used throughout the remainder of the experiment. However, using the ratings provided during the determination of moderate pain, we recorded 2 different temperature stimuli: a moderate pain temperature (40-50 VAS rating) and a high pain temperature (60-70 VAS rating). These 2 temperatures were then deceptively applied to the different cream sites throughout the remainder of sessions 1 and 2, such that the high-temperature stimulus was delivered to the site on which “capsaicin” cream had been applied to convince the participant that this was indeed a more painful stimulus.

To increase believability that the creams contained active substances, false labels were attached to the cream bottles and red food colouring was added to the “capsaicin” cream. Creams were then applied to 2 adjacent 3 × 3 cm squares on the volar surface of the participants' right forearm. Ten minutes following the cream application, we conducted 2 rounds of conditioning. Participants were informed to believe they would be receiving 8 identical moderate thermal stimuli to both cream sites and were instructed to report their perceived pain intensity using the VAS. Participants were also asked before each set of stimuli for an average expectation of the pain they would experience, which acted both to measure belief that capsaicin was working to modulate their subjective pain and to reinforce the pain-increasing quality of the cream. As noted above, during the 2 conditioning rounds, we deceptively applied a moderate temperature to the control vaseline site and a high temperature to the nocebo capsaicin site.

### 2.5. Reinforcement and test

At approximately the same time on the following day, sessions 2 and 3 were conducted with participants inside the MRI scanner and consisted of a reinforcement protocol (session 2) and a test protocol (session 3). The creams were reapplied to the volar surface of both left and right forearms, in the same order and locations as session 1, and participants were reminded of the capsaicin's pain-increasing qualities. To ensure that the protocol for conditioning was consistent between subsequent days, despite the change in the immediate environment (now inside the MRI), reinforcement was conducted by applying 4 noxious stimuli at the same moderate and high temperatures used throughout session 1 to participants' left volar forearm. This was performed on the opposite forearm to prevent sensitisation of the testing area (the right volar forearm).

Following reinforcement, we waited 15 minutes for residual pain and sensitivity to dissipate during which time we acquired structural brain scans. Unlike the conditioning and reinforcement phases, during the test phase, we applied *identical moderate temperature stimuli* to both the control vaseline and nocebo capsaicin sites (Fig. 1A). We asked each participant for an average expectation of pain intensity directly before each stimulation series and instructed them to report the pain intensity continuously throughout the duration of the scan using the button box and the projected digital VAS. Visual analogue scale responses were recorded every 0.5 seconds, and values during each pain period were averaged providing a pain intensity for each noxious stimulus period. Each participant received 2 consecutive series of 8 stimuli, with a separate fMRI series collected during each series of stimuli. Each fMRI series began with a 90-second baseline period before the 8 stimuli presentations. The control vaseline site was always stimulated during the first series, and the nocebo capsaicin site was stimulated during the second series.

### 2.6. MRI data acquisition and preprocessing

Brain images were acquired using a whole-body Siemens MAGNETOM 7 Tesla (7T) MRI system (Siemens Healthcare, Erlangen, Germany) with a combined single-channel transmit and 32-channel receive head coil (Nova Medical, Wilmington, MA). Participants were positioned supine with their head in the coil and sponges supporting the head laterally to minimise movement. A T1-weighted anatomical image set covering the whole brain was collected (repetition time 5000 ms, echo time 3.1 ms, raw voxel size 0.73 × 0.73 × 0.73 mm, 224 sagittal slices, scan time 7 minutes). The 2 fMRI acquisitions each consisted of a series of 134 gradient-echo echo-planar measurements using blood oxygen level-dependent (BOLD) contrast, covering the entire brain. Images were acquired in an interleaved collection pattern with a multiband factor of 4 and an acceleration factor of 3 (repetition time 2500 ms, echo time 26 ms; raw voxel size 1.0 × 1.0 × 1.2 mm, 124 axial slices, scan time 5:35 minutes).

Image preprocessing and statistical analyses were performed using SPM12 and custom software. The first 5 volumes of each scan were removed due to excessive signal saturation. The remaining 129 functional images were slice-time and motion corrected, and the resulting 6 directional movement parameters were inspected to ensure that there was not more than 1 mm of linear movement or 0.5 degrees of rotational movement. None of the participants exhibited motion parameters that exceeded these thresholds. Images were then linearly detrended to remove global signal changes. Physiological noise relating to cardiac (frequency band of 60-120 beats per minute +1 harmonic) and respiratory (frequency band of 8-25 breaths per minute +1 harmonic) frequency was removed using the DRIFTER toolbox,^31^ and the 6-parameter movement-related signal changes were modelled and removed using a linear modelling procedure based on realignment parameters. Each individual's fMRI image sets were then coregistered to their own T1-weighted anatomical, and the T1 was then spatially normalised to the MNI152 template in the Montreal Neurological Institute (MNI) space with these parameters applied to the fMRI image sets. The normalised fMRI images were then spatially smoothed using a 6-mm full width at half maximum Gaussian filter.

### 2.7. Dichotomizing nocebo hyperalgesia responder and nonresponder groups

Participants were grouped as either responders or nonresponders to placebo analgesia based on a bootstrapped permutation procedure.^3^ In brief, mean VAS ratings for each of the 8 noxious stimuli delivered during the control-stimulated series were entered into a permutation model, where 10,000 artificial samples were generated with replacement. This artificial sample was significance tested against 10,000 artificial samples generated from the VAS ratings for each of the 8 noxious stimuli delivered during the nocebo “capsaicin”-stimulated series. If the mean difference between the 2 series was significant, with the “capsaicin” significantly higher than the control, a participant was considered a responder. If not, they were considered a nonresponder. Significant differences between groups with respect to expected changes in pain intensities immediately before testing were determined using paired *t*-tests (2-tailed, *P* < 0.05). Since participants were grouped into either responder or nonresponder categories, we did not assess significant differences between groups for the perceived pain intensity changes. A single factor ANOVA (*P* < 0.05) was used to determine whether there were differences in the temperature applied or pain intensity ratings reported between responder and nonresponder groups during the control-stimulated series to ensure that any reported nocebo hyperalgesia effects did not relate to baseline thermal sensitivity.

#### 2.7.1. Functional magnetic resonance imaging signal intensity statistical analysis

To determine significant changes in signal intensity during each noxious stimulation period, a repeating boxcar model convolved with a canonical hemodynamic response function was applied to both fMRI series. Within this model, scanning volumes overlying stimulus plateau periods were assigned a value of 1, and interstimulus intervals and the initial 90-second baseline period were assigned a value of 0. The resulting contrast images for the responder group (n = 14) were entered into a paired, second-level random-effects analysis to determine significant changes in signal intensity during the capsaicin-cream scan compared with the lidocaine cream scan (*P* < 0.005, uncorrected with a cluster extent threshold of 20 contiguous voxels). Cluster-level correction for multiple comparisons was performed on resulting clusters (*P* < 0.05) to reduce the likelihood of type I errors. The locations of significant clusters in MNI space were tabulated and significant clusters were overlaid onto a rendered view of an individual T1-weighted anatomical image. For each significant cluster, signal intensity changes (β-values) were extracted from the control (vaseline) and nocebo (“capsaicin”) cream scans in both the responder and nonresponder groups to determine the directions of signal changes and whether clusters identified in responders display significant differences in nonresponders. Whilst we did not determine significant differences in β-values extracted from the responder group, to avoid “double dipping,” we did assess the significance between vaseline and “capsaicin” cream scan β-values in the nonresponder group (*P* < 0.05, paired *t*-tests).

#### 2.7.2. Lateral periaqueductal gray matter connectivity statistical analysis

Previously, we identified through brainstem-specific analyses a region of the caudal lPAG ipsilateral to the side of stimulation as primarily responsible for nocebo hyperalgesia.^11^ Using a 1-mm radius sphere placed at that precise lPAG location in full field-of-view images (MNI coordinate X = +2, Y = −30, Z = −5), we first extracted signal intensity change differences (capsaicin—vaseline β-values) in this region to confirm that nocebo hyperalgesia responsiveness was associated with altered activity change within this midbrain pain-modulatory site. Once confirmed, we then used this lPAG sphere as a seed and performed 3 different analyses:

(1) *Forebrain-PAG stimulus-independent connectivity changes* were assessed by conducting a functional connectivity (FC) analysis. This analysis generates contrast images which include the time series of the lPAG seed as a regressor, independent of the timing of noxious stimuli applied. As such, this analysis reveals forebrain regions contacting the PAG during the entire scan period including the baseline anticipation, ramp, pain plateau, and interstimulus interval periods. Using these contrast images, a random-effects paired, voxel-by-voxel analysis was conducted in the responder group comparing altered lPAG coupling between the control vaseline- and capsaicin-cream fMRI series (*P* < 0.005, uncorrected with a cluster extent threshold of 20 contiguous voxels). The locations of significant clusters in MNI space were tabulated, and significant clusters were overlaid onto a rendered view of an individual T1-weighted anatomical image. We applied small volume correction (*P* < 0.05) to reduce the likelihood of type II errors. From the resulting clusters, β-values representing stimulus-independent connectivity with the lPAG in each series were extracted from both the vaseline- and capsaicin-cream scans in responder and nonresponder groups. We determined whether these changes in connectivity were significantly different between vaseline- and capsaicin-cream scans in the nonresponder group (*P* < 0.05, paired *t*-tests).
(2) *Forebrain-lPAG stimulus-dependent connectivity changes* in nocebo responder and nonresponder groups were assessed by conducting a psychophysiological interaction (PPI) analysis. This involves extracting the time series of the lPAG from each participant's vaseline- and capsaicin-cream scans and convolving it with the repeating boxcar model which isolates scan periods in which the plateau period of noxious stimuli occurred. This generates a new stimulus × lPAG time-series regressor which is then applied to functional series to create new contrast images of stimulus-dependent lPAG connectivity. Using these contrast images, a random-effects paired, voxel-by-voxel analysis was conducted in responders comparing the vaseline- and capsaicin-cream scans (*P* < 0.005, uncorrected with a cluster extent threshold of 20 contiguous voxels). We applied small volume correction (*P* < 0.05) to reduce the likelihood of type II errors. The locations of significant clusters in MNI space were tabulated, and significant clusters were overlaid onto a rendered view of an individual T1-weighted anatomical image. From the resulting clusters, β-values representing stimulus-dependent connectivity with the lPAG in each series were extracted from both the vaseline- and capsaicin-cream scans in responder and nonresponder groups. We determined whether these changes in connectivity were significantly different between vaseline- and capsaicin-cream scans in the nonresponder group (*P* < 0.05, paired *t*-tests).
(3) *Network properties and directed connectivity* in PPI and FC clusters were compared by conducting 2 separate dynamic causal modelling (DCM) analyses. Dynamic causal modelling is a technique in which cluster time series are compared with determine whether the activity of 1 region over time can predict the activity in a second, connected region. We conducted our 2 DCMs using the following parameters: slice timing = 1.25 seconds (modelled to the centre slice of acquisition), echo time = 0.026 seconds, bilinear modulatory effects, 1 state per region, stochastic effects off, centred inputs on, and a time-series fit. The timings of noxious stimuli were modelled specifically in the PPI DCM and added as potential contributors to all extrinsic and intrinsic connections of the full model due to the inherent stimulus dependency of these clusters. After identifying the optimal reduced model through nested search, individual participant parameter estimates for each resulting between-cluster and self-connection were extracted and effect sizes and 95% confidence intervals were calculated by Cohen d to identify directed connections with medium-to-large effects between nocebo responders and nonresponders (Cohen d > 0.5). Posterior probabilities of the reduced model after nested search were threshold at *P* > 0.95, and effect sizes of parameter estimate differences between responders and nonresponders were discerned using Cohen d tests.

### 2.8. Psychometric involvement in nocebo hyperalgesia expression and top-down upper midbrain recruitment

Each participant's questionnaire values were scored using the validated scoring criteria for each administered questionnaire and then tabulated. Mean differences between responder and nonresponder groups were significance tested using 2-sample *t*-tests. In addition, linear regression analyses were performed using both nocebo ability and signal intensity change within the lPAG to determine whether variance in these scores held predictive capacity over either the magnitude of nocebo hyperalgesia expressed or the top-down activation of pain modulatory midbrain systems.

#### 2.8.1. Data availability

Anonymised data files may be made available to qualified investigators upon request.

## 3. Results

### 3.1. Psychophysics

Analysis of perceived pain intensities revealed that 14 of the 25 participants displayed a significant increase in perceived pain intensity during stimulation of the capsaicin cream compared with the vaseline cream site (mean ± SEM VAS: vaseline 39.09 ± 2.15*,* capsaicin 57.19 ± 2.33, *P* < 0.001). In the remaining 11 participants, mean pain intensities during vaseline- vs capsaicin-cream stimulations were not significantly different (mean ± SEM VAS: Vaseline 42.47 ± 4.27*,* capsaicin 46.23 ± 4.04, *P* = 0.15) (Figs. 1B and C). Despite these differences, both the responder and nonresponder groups expected pain intensity to increase significantly during the capsaicin- compared with the vaseline cream scans (mean ± SEM VAS expectation responder: vaseline 47.69 ± 1.00, capsaicin 70.00 ± 2.34, *P* < 0.001; nonresponder: vaseline 49.44 ± 1.66, capsaicin 63.89 ± 1.98, *P* < 0.001) (Fig. 1C). Mean VAS rating during the vaseline cream series did not significantly differ between groups (*P* = 0.47) nor the elected moderate temperature applied to the vaseline and capsaicin cream sites during the test phase whilst functional series were collected (mean ± SEM °C: responder 46.96 ± 0.29, nonresponder 47.00 ± 0.26, *P* = 0.93). Consistent with our previous study, extraction of β-values from the lPAG sphere revealed a significant positive linear interaction between nocebo ability (change in mean VAS between vaseline- and capsaicin scans) and change in lPAG β-value. That is, greater nocebo hyperalgesic responses were associated with greater change in activation within the lPAG (R = 0.41; *P* = 0.03) (Fig. 1D).

### 3.2. Nocebo-related forebrain signal intensity changes

**Figure 2.**
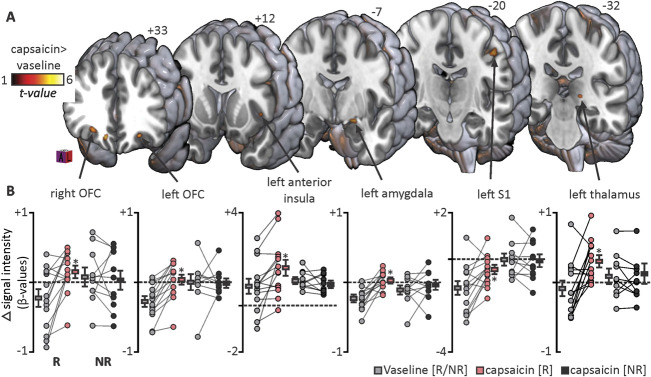
Main effect of nocebo-related forebrain activity change. (A) Areas of significant signal intensity increases during noxious stimulation of nocebo “capsaicin” relative to vaseline cream sites in nocebo hyperalgesia responders overlaid onto a rendered MNI152 template. Signal intensity increases are hot colour-coded and slice locations in the Montreal Neurological Institute space and are indicated at the top of each section. (B) Individual participant and group mean β-values extracted from significant clusters for both responder and nonresponder groups. Lines connecting individual grey, red, and dark grey circles indicate an individual's paired activity change. *P < 0.001; voxel-by-voxel analysis. NR, nonresponder; OFC, orbitofrontal cortex; R, responder; S1, primary somatosensory cortex.

Noxious stimulus-evoked signal intensity changes revealed that nocebo hyperalgesia was associated with signal increases in a number of forebrain regions. Responders displayed significant signal increases during the capsaicin compared with the vaseline cream scans in the bilateral orbitofrontal cortices (OFCs), the left (contralateral to stimulus side) anterior insula, amygdala, thalamus, and in the region of the primary somatosensory cortex (S1) that represents the arm (Fig. 2A). In no forebrain region did signal intensity decrease during capsaicin compared with vaseline cream scans in nocebo responders. Extraction of β-values from each significant cluster revealed that whilst signal intensity increased during capsaicin compared with vaseline cream scans in the responder group, no significant signal changes occurred in the nonresponder group in any cluster (Fig. 2B, Table 1).

**Table 1:** Location, significance level, and cluster size of brain regions significantly altered in activation during nocebo hyperalgesia expression relative to typical pain processing.

	MNI coordinates			t-value	Cluster size	Responders β-values (mean ± SEM)		Nonresponders β-values (mean ± SEM)	
	X	Y	Z			Vaseline	Capsaicin	Vaseline	Capsaicin
Capsaicin > vaseline									
Right OFC	26	32	−13	3.68	130	−0.24 ± 0.11	0.16 ± 0.08*	0.08 ± 0.11	0.04 ± 0.12
Left OFC	−25	33	−13	3.88	147	−0.28 ± 0.06	0.04 ± 0.07*	−0.01 ± 0.09	0.01 ± 0.05
Left anterior insula	−43	12	1	3.56	35	0.86 ± 0.29	1.65 ± 0.30*	1.09 ± 0.11	0.94 ± 0.11
Left amygdala	−20	−7	−11	4.81	156	−0.25 ± 0.04	0.04 ± 0.03*	−0.11 ± 0.06	−0.05 ± 0.06
Left S1	−46	−20	45	4.06	172	−1.28 ± 0.26	−0.48 ± 0.17*	−0.06 ± 0.22	−0.11 ± 0.23
Left thalamus	−18	−28	1	3.46	35	−0.09 ± 0.10	0.31 ± 0.07*	0.10 ± 0.09	0.13 ± 0.13

Coordinates are in the Montreal Neurological Institute (MNI) space. Cluster sizes are derived from resliced 1-mm isotropic image series.

* *P* < 0.005 voxel-by-voxel analysis capsaicin > vaseline.

OFC, orbitofrontal cortex; S1, primary somatosensory cortex.

### 3.3. Stimulus-dependent lateral periaqueductal gray matter connectivity changes

**Figure 3.**
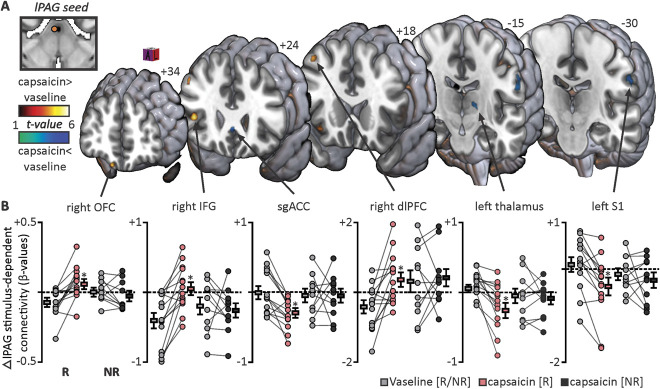
Forebrain changes in brainstem midbrain coupling during noxious stimulus periods. (A) Areas in which connectivity with the lateral midbrain periaqueductal gray matter (lPAG) was significantly altered during noxious stimuli between the vaseline and nocebo “capsaicin” scans overlaid onto a rendered MNI152 template. Connectivity increases and decreases are indicated by the hot and cool colour scales, respectively. Slice location is in the Montreal Neurological Institute space and is indicated at the top of each section. (B) Individual participant and group-level β-values extracted from significant clusters for nocebo responders and nonresponders. Lines connecting individual grey, red, and dark grey circles indicate an individual's connectivity changes. *P < 0.001; voxel-by-voxel analysis. dlPFC, dorsolateral prefrontal cortex; IFG, inferior frontal gyrus; NR, nonresponder; OFC, orbitofrontal cortex; R, responder; S1, primary somatosensory cortex; sgACC, subgenual cingulate cortex.

Psychophysiological interaction analysis revealed a number of forebrain sites which either significantly increased or decreased noxious stimulus-related lPAG connectivity during capsaicin compared with vaseline cream scans in responders. Increased lPAG connectivity occurred in the right OFC, right inferior frontal gyrus (IFG), and right dorsolateral prefrontal cortex (dlPFC). Conversely, decreased lPAG coupling occurred in the left thalamus, left S1, and subgenual anterior cingulate cortex (sgACC) (Fig. 3A). Similar to activation parameters, for each of these forebrain clusters, extraction of β-values from the nonresponder group revealed no significant lPAG connectivity changes (Fig. 3B, Table 2).

**Table 2:** Location, significance level, and cluster size of brain regions in which lateral midbrain periaqueductal gray matter stimulus-evoked connectivity changes were significantly different between nocebo “capsaicin” and control vaseline cream scans.

	MNI coordinates			t-value	Cluster size	Responders β-values (mean ± SEM)		Nonresponders β-values (mean ± SEM)	
	X	Y	Z			Vaseline	Capsaicin	Vaseline	Capsaicin
Capsaicin > vaseline									
Right OFC	13	50	−25	4.47	94	−0.08 ± 0.02	0.06 ± 0.03*	0.01 ± 0.03	−0.03 ± 0.0.03
Right IFG	43	24	3	5.93	204	−0.41 ± 0.10	0.06 ± 0.08*	−0.20 ± 0.11	−0.26 ± 0.09
Right dlPFC	44	18	47	4.06	114	−0.53 ± 0.18	0.31 ± 0.20*	0.35 ± 0.29	0.46 ± 0.23
Capsaicin < vaseline									
Left sgACC	3	22	−4	5.80	75	0.01 ± 0.08	−0.31 ± 0.05*	−0.06 ± 0.08	−0.06 ± 0.09
Left thalamus	−13	−15	8	4.16	95	0.06 ± 0.04	−0.25 ± 0.09*	−0.06 ± 0.09	−0.10 ± 0.07
Left S1	−60	−14	34	4.62	275	0.13 ± 0.14	−0.37 ± 0.17*	−0.11 ± 0.11	−0.25 ± 0.14

Coordinates are in the Montreal Neurological Institute (MNI) space. Cluster sizes are derived from resliced 1-mm isotropic image series.

* *P* < 0.005 voxel-by-voxel analysis capsaicin > vaseline.

dlPFC, dorsolateral prefrontal cortex; IFG, inferior frontal gyrus; OFC, orbitofrontal cortex; S1, primary somatosensory cortex; sgACC, subgenual anterior cingulate cortex.

### 3.4. Stimulus-independent lateral periaqueductal gray matter connectivity changes

**Figure 4.**
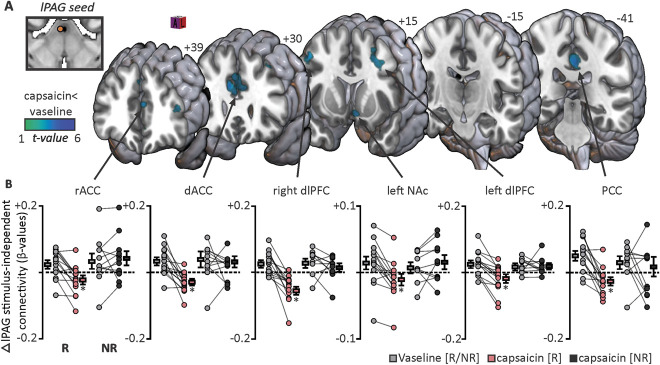
Forebrain changes in brainstem midbrain coupling independent of noxious stimulus periods. (A) Areas in which connectivity with the lateral midbrain periaqueductal gray matter (lPAG) were significantly altered over the entire scan between vaseline and nocebo “capsaicin” sites overlaid onto a rendered MNI152 template. Connectivity decreases are indicated by the cool colour scale. Slice location is in the Montreal Neurological Institute space and is indicated at the top of each section. (B) Individual participant and group-level β-values extracted from responder and nonresponder groups. Lines connecting individual grey, red, and dark grey circles indicate an individual's connectivity changes. *P < 0.001; voxel-by-voxel analysis. dACC, dorsal anterior cingulate cortex; dlPFC, dorsolateral prefrontal cortex; NAc, nucleus accumbens; NR, nonresponder; PCC, posterior cingulate cortex; R, responder; rACC, rostral anterior cingulate cortex.

Functional connectivity analysis also revealed a number of forebrain sites which displayed significantly decreased noxious stimulus-independent (ie, connectivity over the entire scan) lPAG connectivity during capsaicin compared with vaseline cream scans in responders. Decreases in lPAG connectivity during capsaicin compared with vaseline scans occurred in the bilateral dlPFC, left nucleus accumbens (NAc), midline rostral (rACC), midline dorsal (dACC), and midline posterior (PCC) cingulate cortices (Fig. 4A). In no forebrain region was connectivity greater during the capsaicin-cream scan compared with the vaseline cream scan. Again, for each of these forebrain clusters, extraction of β-values from the nonresponder group revealed no significant lPAG connectivity changes (Fig. 4B, Table 3).

**Table 3:** Location, significance level, and cluster size of brain regions in which lateral midbrain periaqueductal gray matter stimulus-independent connectivity changes were significantly different between nocebo “capsaicin” and control vaseline cream scans.

	MNI coordinates			t-value	Cluster size	Responders β-values (mean ± SEM)		Nonresponders β-values (mean ± SEM)	
	X	Y	Z			Vaseline	Capsaicin	Vaseline	Capsaicin
Capsaicin < vaseline									
Bilateral rACC	−2	39	17	4.96	103	0.26 ± 0.11	−0.24 ± 0.11*	0.35 ± 0.21	0.43 ± 0.20
Bilateral dACC	0	30	30	5.54	1763	0.35 ± 0.10	−0.32 ± 0.08*	0.30 ± 0.16	0.17 ± 0.11
Right dlPFC	54	17	27	6.27	1087	0.28 ± 0.09	−0.44 ± 0.10*	0.10 ± 0.12	0.17 ± 0.18
Left dlPFC	−24	33	25	5.66	269	0.10 ± 0.15	−0.49 ± 0.09*	0.30 ± 0.11	0.16 ± 0.11
Left NAc	−6	9	−12	4.88	111	0.15 ± 0.15	−0.11 ± 0.08*	0.08 ± 0.12	0.17 ± 0.18
Bilateral PCC	−2	−41	35	8.34	1655	0.52 ± 0.12	−0.29 ± 0.10*	0.31 ± 0.16	0.01 ± 0.02

Coordinates are in the Montreal Neurological Institute (MNI) space. Cluster sizes are derived from resliced 1-mm isotropic image series.

* *P* < 0.005 voxel-by-voxel analysis capsaicin > vaseline.

dACC, dorsal anterior cingulate cortex; dlPFC, dorsolateral prefrontal cortex; NAc, nucleus accumbens; PCC, posterior cingulate cortex; rACC, rostral anterior cingulate cortex.

### 3.5. Lateral periaqueductal gray matter connectivity direction analysis

**Figure 5.**
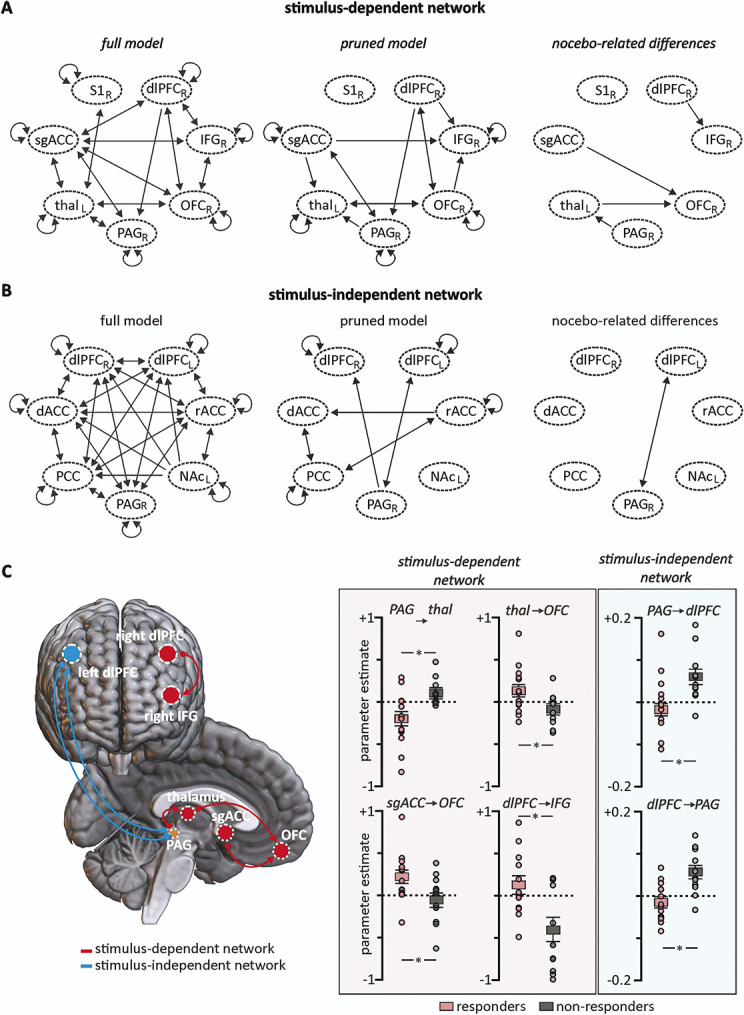
Defining a stimulus-dependent and -independent network of nocebo-related brainstem midbrain activity. Dynamic causal modelling (DCM) was conducted on both stimulus-dependent and stimulus-independent networks by entering individual participant time series of significant clusters as well as the lateral periaqueductal gray matter (PAG) seed into 2 separate full model designs. Each anatomically possible connection was turned on, and the timing of stimuli was modelled in only the psychophysiological interaction DCM to account for the stimulus dependency of these connections. After specifying and estimating the full models, the nested search was conducted to construct a pruned model, or, only those directed connections that significantly added to optimal model evidence. Full and pruned models are displayed in the left and middle panels of (A and B), respectively. Pruned models were threshold at connections with a posterior parameter estimate *P* > 0.95. Each first-level model was then inspected for each participant, and individual connection parameter estimates were extracted. Two-sample *t*-tests were conducted comparing mean parameter estimates between responders and nonresponders. (C) Rendered view of directed functional connections displaying significant group-level differences between nocebo responders and nonresponders, accompanied by individual and group-level plots of the significance of each of these 6 connections. Connections within the psychophysiological network with the PAG are displayed in red lines and red-shaded panel, and those in the functional connectivity network are displayed in blue lines and shaded panel. dlPFC, dorsolateral prefrontal cortex; IFG, inferior frontal gyrus; OFC, orbitofrontal cortex; PAG, midbrain periaqueductal gray matter; sgACC, subgenual anterior cingulate cortex; thal, thalamus.

To determine the directionality of signalling between sites uncovered in either our PPI or FC analyses (ie, whether regions within either system were driving output or receiving information from the lPAG), 2 DCM analyses were performed with model timings added to the PPI network to establish their specific engagement within stimulus periods. A full model was first constructed comprising each anatomically possible connection in either network (Figs. 5A and B) before the nested search was conducted to prune connections until optimal model free energy was reached—ie, connections were only retained if the time series of 1 region could predict another within the constraints of the full model in either a forward, backward, or reciprocal direction (Figs. 5A and B). For the stimulus-dependent lPAG connections, nocebo hyperalgesia was driven by ascending input from the lPAG to the contralateral thalamus, with both the thalamus and sgACC then feeding into the ipsilateral OFC. In addition, connectivity from the right dlPFC to the right IFG was also different between responder and nonresponder groups (Fig. 5C, Table 4). Conversely, the stimulus-independent system displayed 2 significantly different connections, a reciprocal connection between the lPAG and the left dlPFC (Fig. 5C, Table 4).

**Table 4:** Significant modulatory parameter estimate values determined by nested search dynamic causal modelling.

	Mean (±SEM) responder	Mean (±SEM) nonresponder	Cohen d (effect size) 95% CI
Stimulus-dependent connectivity			
PAG → left thalamus	−0.20 ± 0.08	0.11 ± 0.05	1.95 (0.30-2.01)
left thalamus → right OFC	0.13 ± 0.03	−0.01 ± 0.04	1.25 (0.05-1.70)
sgACC → right OFC	0.22 ± 0.07	−0.05 ± 0.08	0.97 (0.11-1.77)
right dlPFC → right IFG	0.12 ± 0.11	−0.42 ± 0.16	0.98 (0.18-1.85)
Stimulus-independent connectivity			
left dlPFC → PAG	−0.02 ± 0.01	0.06 ± 0.02	1.42 (0.58-2.35)
PAG → left dlPFC	−0.02 ± 0.01	0.06 ± 0.02	1.30 (0.51-2.27)

Effect sizes were calculated by Cohen d.

dlPFC, dorsolateral prefrontal cortex; IFG, inferior frontal gyrus; OFC, orbitofrontal cortex; PAG, midbrain periaqueductal gray matter; sgACC, subgenual anterior cingulate cortex.

### 3.6. Nocebo-related psychological differences and lateral periaqueductal gray matter activation prediction

No significant differences between groups nor predictive capacity against the magnitude of nocebo hyperalgesia expressed were identified in any of the questionnaires administered as part of this investigation (Table 5). However, we identified a significant positive linear relationship between behavioural inhibition scores and signal intensity change within the lPAG (R = 0.65; *P* < 0.001) (Table 5). That is, those participants more sensitive to potential punishment and uncertainty and more likely to show risk-aversive tendencies showed greater activation of the lPAG under nocebo conditions and vice versa in those with lower recorded scores.

**Table 5:** Group mean differences and linear interactions in psychological measures, behavioural expression of nocebo hyperalgesia, and signal intensity change within the lateral midbrain periaqueductal gray matter.

	Mean (±SEM) responder	Mean (±SEM) nonresponder	*P*	Nocebo ability R-value	Nocebo ability *P*	ΔlPAG SI R-value	ΔlPAG SI *P*
BAS	41.07 ± 1.25	43.7 ± 1.06	0.17	−0.01	0.98	+0.03	0.87
BIS	21.00 ± 1.09	20.30 ± 1.23	0.69	+0.19	0.38	+0.65*	<0.001*
STAI-S	44.07 ± 0.07	44.18 ± 0.89	0.89	+0.12	0.58	+0.05	0.81
STAI-T	45.79 ± 1.02	45.36 ± 1.15	0.79	+0.31	0.13	+0.29	0.16
LOT-R	12.43 ± 0.64	12.89 ± 0.83	0.69	+0.15	0.49	−0.15	0.51
PCS	18.57 ± 2.33	14.20 ± 1.99	0.22	−0.02	0.96	+0.29	0.17

* Significant at *P* < 0.05 statistical threshold.

Δ, change in; BAS, behavioural activation scale; BIS, behavioural inhibition scale; LOT-R, life orientation test revised form; lPAG, lateral column of the midbrain periaqueductal gray matter; PCS, pain catastrophizing scale; SI, signal intensity; STAI-S, state component of the state and trait anxiety inventory; STAI-T, trait component of the state and trait anxiety inventory.

## 4. Discussion

Our investigation records several key findings surrounding the brain systems associated with human expression of the nocebo hyperalgesia phenomenon. Increased activation was primarily observed in the OFC, insula, and amygdala. Additionally, PPI analysis revealed stimulus-dependent connectivity changes between the lPAG and the OFC, ACC and dlPFC, while FC analysis revealed stimulus-independent lPAG connectivity with the ACC, dlPFC and NAc associated with greater nocebo hyperalgesia. Whilst areas such as the ACC, dlPFC, and NAc are also involved in generating placebo analgesia responses, our analysis suggests that during nocebo hyperalgesia, the dlPFC directly drives the lPAG, whereas during placebo analgesia, the ACC drives lPAG signal changes.

Whether nocebo and placebo effects are mediated by the same neural networks has been a matter of debate. We have previously shown that at least at the brainstem level, there appears to be a consistent set of structures, including the PAG-RVM circuit, that mediate both pain modulatory effects.^11^ With regard to higher brain regions, it has been previously shown that nocebo hyperalgesia is associated with altered fMRI signal intensity changes in the insula, ACC, orbital prefrontal cortex (OFC), and amygdala.^25,36,37,43^ Consistent with these previous studies, we also found nocebo-related signal intensity changes in the anterior insula, OFC, and amygdala, although we did not find signal changes in the ACC. Whilst we have previously reported that placebo analgesia was also associated with signal changes in the OFC and amygdala, we also found placebo analgesia-related changes in the ACC, ventrolateral prefrontal cortex, and dlPFC.^12^ This overall pattern of cortical engagement combined with our previous brainstem results suggests that despite some overlapping circuitry, nocebo and placebo evoke changes in pain perception that recruit different higher-order cognitive brain structures. Indeed, in a previous study in which placebo and nocebo interventions were directly compared, it was reported that nocebo activated the insula, OFC, and the PAG, whereas placebo preferentially recruited the striatum.^19^ Whilst this previous report is not entirely consistent with our and other findings, it does show that at least part of the neural circuitry underpinning nocebo and placebo are different.

Consistent with this idea, we found differential lPAG connectivity changes during nocebo hyperalgesia compared with our previous report of lPAG connectivity during placebo analgesia. We found both lPAG-dlPFC and lPAG-ACC stimulus-dependent and -independent connectivity's change during nocebo hyperalgesia, despite not finding signal intensity changes in these 2 regions. More specifically, lPAG-ACC stimulus-dependent and -independent connectivity decreased during nocebo, whereas we previously showed that during placebo analgesia they increased.^10^ Similarly, we found lPAG-dlPFC stimulus-independent connectivity decreased during nocebo hyperalgesia but increased during placebo analgesia, and only during nocebo did we find lPAG-dlPFC stimulus-independent changes. Although overall we found similar lPAG connectivity changes with the ACC and dlPFC during nocebo and placebo paradigms, DCM analysis revealed that the lPAG was driven primarily by the left dlPFC during nocebo hyperalgesia, whereas it was driven primarily by the ACC during placebo analgesia. Decreased or negative stimulus-independent connectivity occurs in brain networks whose signal intensity fluctuations are diametrically opposed^18^ and may indicate active opposition of 1 brain region's function on another, or that 1 region is activating nearby inhibitory neurons. Here, we suggest that under successful induction of nocebo hyperalgesia, the lPAG begins to work in this anticorrelated pattern with both the dlPFC and ACC, 2 sites previously demonstrated to strengthen in functional coupling under the inverse pain modulatory phenomenon, placebo analgesia.^7,26^ These findings suggest that whilst a number of forebrain regions display altered connectivity patterns during nocebo and placebo, these areas appear to be differentially engaged during the 2 paradigms in which perceived pain either increases or decreases.

In contrast to placebo analgesia in which we found stimulus-dependent lPAG-NAc connectivity increases, during nocebo hyperalgesia we found stimulus-independent lPAG-NAc connectivity decreases. The NAc forms part of the ventral striatum and contacts the prefrontal cortex to drive reward anticipation, decision making, and error predictions.^2^ Correcting perception-anticipation differentials is a critical component in mounting placebo responses,^2,39^ and our results show that changes in ongoing coupling between the NAc and lPAG are also associated with nocebo hyperalgesic responses. Interestingly, whilst we previously found that placebo analgesia responses were associated with ongoing coupling between the lPAG and the amygdala, we found no such coupling during nocebo hyperalgesia, although we did find signal intensity increases in the amygdala, in the region of the central nucleus, which we did not find during placebo analgesia. It is well established that the amygdala plays a critical role in pain processing and aversive learning, with multiple studies showing that increasing amygdala activity can generate or facilitate pain-like behaviours.^28,46^ It has also been shown that the central amygdala projects to the lPAG, and it is possible that amygdala signal changes mediate pain intensity changes through this projection.^1^

Furthermore, our DCM analyses revealed that the important connection driving signal changes in the lPAG during nocebo hyperalgesia was the left dlPFC-lPAG. Interestingly, this analysis revealed that an ongoing reciprocal coupling between the dlPFC and lPAG was important, suggesting that the dlPFC was receiving both ongoing ascending inputs from, and sending ongoing descending inputs to the lPAG. By contrast, we previously showed that during placebo analgesia, ongoing descending projections to the lPAG from the hypothalamus and the ACC were critical in driving lPAG signal changes, although we did also identify left dlPFC-lPAG ongoing connectivity changes during placebo analgesia. It has been shown that dlPFC is involved in mediating placebo analgesia as well as expectations, decision making, and error prediction.^22,30,34,48^ Consistent with these findings, it was recently shown that the dlPFC demonstrates reduced activation, along with altered coupling patterns with pain processing areas such as the posterior insula, primary somatosensory cortex, and PAG in individuals who express greater variation in perceived pain intensity during a series of identical noxious stimuli.^9^ Furthermore, transient direct current stimulation of the dlPFC blunts nocebo hyperalgesia and boosts placebo analgesia responses.^45^ Given these findings, it is likely that the lPAG is driven by inputs from the dlPFC to modulate incoming noxious information.

In addition, we found stimulus-dependent connections from the lPAG to higher brain regions involved in nocebo hyperalgesia. We found noxious stimulus-driven changes in lPAG connectivity with the ventroposterior lateral thalamus as well as between the thalamus and sgACC with the OFC during nocebo hyperalgesia. The OFC has been widely implicated in emotional and decision-making behaviours^21,38^ and receives highly integrated sensory information.^5^ It has been reported that whilst brain regions such as the primary somatosensory cortex code absolute stimulus intensity, activity in the OFC reflects the relative rank of pain and overall value.^50^ In addition, the sgACC is considered a core interface between cognition, emotion, and pain.^45,47^ Previous investigations have established connectivity between the sgACC and PAG driving pain signals and threat responses in humans, and our imaging data combined with the only psychological finding of significance suggesting those more sensitive to threat responses and risk-aversive behaviours (ie, greater BIS scores) suggest that this region receives information from the lPAG as part of endogenously encoded pain enhancement by nocebo hyperalgesia intervention.^8,51^ Given this, it is likely that the activity and connectivity changes in the OFC reflect a point of convergence from ascending input from the lPAG to subnuclei of the thalamus and cingulate cortices, manifesting as individuals heightening their relative ranking of painful stimuli between the first and second series of stimuli.

It is important to note some limitations. First, it was not possible to counterbalance the design such that the vaseline cream site was always stimulated first and the capsaicin second. This ordering effect could have introduced sensitization and or habituation effects. However, since only approximately half of the participants displayed a hyperalgesic response, we suggest that a significant ordering effect is unlikely. Second, functional analyses were threshold at *P* < 0.005, uncorrected for multiple comparisons. To reduce the chances of type II errors, we implemented a minimum cluster threshold of 20 contiguous voxels. Third, our stimulus-independent connectivity analysis involved assessing lPAG connectivity over the entire scanning period which included periods of noxious stimulation. Whilst signal coupling may have been influenced by overall signal intensity change, we suggest that this effect would be minimal since the stimulus periods were less than 25% of the total scan. Finally, despite satisfying apriori power analyses, our sample size was limited to 25 pain-free participants. It would be of benefit to establish these regional activation and connectivity patterns within a larger cohort, across a longitudinal induction of nocebo hyperalgesia, or within chronic pain states to determine whether these nodes of cognitive and pain modulatory systems hold critical functions in these alternate experimental designs.

By combining activation and connectivity measures, this investigation demonstrates that nocebo hyperalgesia is associated with signal and connectivity changes in multiple forebrain regions. One region, the dlPFC, appears to be critical in driving upper brainstem pain modulatory circuitry to produce increases in perceived pain by means of response conditioning to a nocebo substance. Given that the dlPFC lends itself to modulation by electrical or magnetic stimulation, it may well be possible to modulate the pain response by noninvasive dlPFC stimulation.

## Conflict of interest statement

The authors have no conflicts of interest to declare.
